# ResOpsUS, a dataset of historical reservoir operations in the contiguous United States

**DOI:** 10.1038/s41597-022-01134-7

**Published:** 2022-02-03

**Authors:** Jennie C. Steyaert, Laura E. Condon, Sean W.D. Turner, Nathalie Voisin

**Affiliations:** 1 Department of Hydrology and Atmospheric Sciences, University of Arizona, Tucson, USA; 2 Pacific Northwest National Laboratory, Richland, USA; 3 Department of Civil and Environmental Engineering, University of Washington, Seattle, USA

**Keywords:** Hydrology, Environmental impact

## Abstract

There are over 52,000 dams in the contiguous US ranging from 0.5 to 243 meters high that collectively hold 600,000 million cubic meters of water. These structures have dramatically affected the river dynamics of every major watershed in the country. While there are national datasets that document dam attributes, there is no national dataset of reservoir operations. Here we present a dataset of historical reservoir inflows, outflows and changes in storage for 679 major reservoirs across the US, called ResOpsUS. All of the data are provided at a daily temporal resolution. Temporal coverage varies by reservoir depending on construction date and digital data availability. Overall, the data spans from 1930 to 2020, although the best coverage is for the most recent years, particularly 1980 to 2020. The reservoirs included in our dataset cover more than half of the total storage of large reservoirs in the US (defined as reservoirs with storage greater 0.1 km^3^). We document the assembly process of this dataset as well as its contents. Historical operations are also compared to static reservoir attribute datasets for validation.

## Abstract (summary)

**Table:** 

Measurement(s)	storage • inflow • outflow • evaporation • elevation
Technology Type(s)	digital curation
Factor Type(s)	temporal interval
Sample Characteristic - Environment	reservoir
Sample Characteristic - Location	contiguous United States of America

Machine-accessible metadata file describing the reported data: 10.6084/m9.figshare.17161415

## Background & Summary

There are more than 52,000 dams^1^ across the Contiguous United States. They regulate all major rivers^2,3^ and provide a total storage capacity of roughly 75% of the mean annual CONUS runoff^4^. The impact of these dams on human systems and the natural environment is substantial. By regulating the flow of rivers, dams provide reliable water supply for various sectors (agriculture, power, and public supply), enable year-round navigation, protect communities against damaging floods, and support regional economic development^5–11^. Dams also cause a range of undesirable impacts, including disruption in water quality patterns through temperature, dissolved oxygen, and sediment regime changes, leading to loss of original biodiversity in affected river systems^12–15^. Despite the critical role that dams play in watersheds across the US, we lack a standardized multi-agency dataset of historical reservoir operations in the United States.

The National Inventory of Dams (NID), the National Anthropogenic Barriers Dataset (NABD) and the Global Reservoirs and Dams (GRanD) provide consistent national scale information on static reservoir properties such as reservoir location, construction, purpose and storage capacity. However, these datasets lack time series records of operations. Records of reservoir operations are held by a plethora of local, state, and federal national agencies, in a variety of formats, and with varying degrees of public accessibility. Many records are not available unless specifically requested through the relevant agency.

The lack of national operations data is a significant limitation for watershed studies. National scale hydrologic simulations generally derive operating policies indirectly from reservoir characteristics and assumptions about streamflow and demand^10,16–18^. The few studies that have utilized historical data to analyse reservoir operations have done so at smaller scales using only select reservoirs due to the lack of a national dataset^10,19–21^.

Here we present a nation-wide dataset of operational records for 679 major dams spread across the Contiguous US (CONUS) and areas draining to the CONUS. Our dataset, *ResOpsUS* is the first of its kind, combining historical records held by 40 different agencies (Bureau of Reclamation, Army Corps of Engineers, California Data Exchange, Water Data for Texas, United States Geological Survey and other independent reservoir operators) in a centralized and standardized dataset. The *ResOpsUS* dataset includes daily inflow, outflow, storage, elevation and evaporation where available. Additionally, ResOpsUS may be used to assess historical reservoir storage and river regulation as well as infer reservoir operations for the large dams in CONUS^22^. This paper summarizes the dataset properties and assembly method.

## Methods

### Data Assembly

**Fig. 1:**
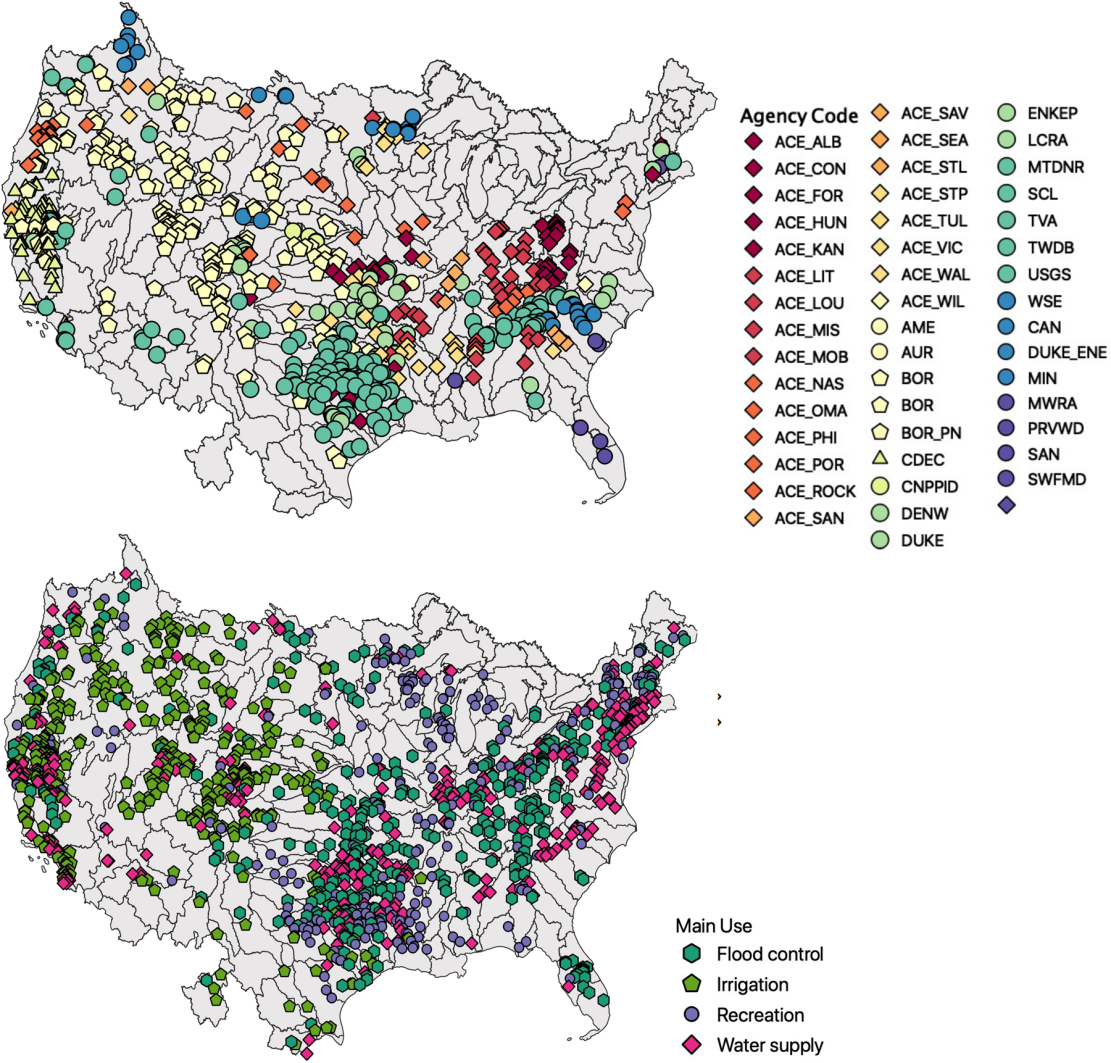
Maps of the reservoirs included in ResOpsUS. Summarized by (**a**) the agencies who contributed data and (**b**) the main purpose of the dams. Different shapes correspond to different regional offices all run by either the Army Corps of Engineers (ACE), the Bureau of Reclamation (BOR), Tennessee Valley Authority (TVA) or California Data Exchange (CDEC), while circles correspond to individual dam operators who are not a part of theses larger organizations.

Our dataset includes dams in CONUS or on rivers in Canada or Mexico that drain to the CONUS. Our goal was to capture as much of the total storage in the country as possible. We first obtained as many records as possible from major agency web portals. Approximately half of the records contained in *ResOpsUS* were obtained in this way, through portals of United States Army Corps of Engineers (USACE), United States Bureau of Reclamation (USBOR), California Data Exchance Center (CDEC), Water Data for Texas, and the United States Geological Survey (USGS) as described in the *agency_attributes.csv* file. The remaining records were collected through direct request to individual agencies. Starting with the largest structures and the largest agencies, we contacted dam operators for daily time series information of inflow, outflow, storage, elevation and evaporation dating to the creation of the dam. There are many agencies that control multiple reservoirs, such as the Bureau of Reclamation, or the Tennessee Valley Authority. In all cases we requested operations data for all of the reservoirs operated by an agency at the time of the request. The dataset therefore includes a handful of reservoirs that fall below the 100 MCM threshold. In total, we received data from 48 agencies across the CONUS as shown in Fig. 1. The data gathering process required multiple Freedom of Information Act (FOIA) requests, emails and phone calls.

### Data processing

To ensure the data are shared in their rawest format, we made no adjustments to the data we received other than to remove duplicate row entries and to make date formatting and units consistent. *ResOpsUS* provides daily storage and evaporation in units of million cubic meters, inflow and release in cubic meters per second, and elevation in meters. Gaps in data are denoted with “NA”. If the agency utilized United States Geological Survey (USGS) stream gages to supplement data for inflow, outflow or elevation, we provided those records in the dataset, as well as the gauge numbers, in the *time_series_inventory.csv* file. Data from the Tennessee Valley Authority was an exception because a list of stream gages was given for all reservoir’s inflow. For TVA, *ResOpsUS* provides just the list of inflow gages. If data were provided by multiple agencies for the same reservoir, we picked the data with the longest period of record and used that as the representative time series. If an agency only gathered elevation data rather than storage data, we also requested storage elevation tables. For these locations we provide estimated storage values using linear interpolations of the storage elevation curves  and denote which storages are linearly interpolated in the *agency_attributes.csv* file.

Finally, we evaluate the dataset by comparing it to reservoir attributes provided in the GRanD database. This analysis is discussed in more detail in section 3.1. While there are multiple differences between these datasets, we did not correct any large differences unless there were obvious erroneous entries in the raw data. Specifically, there were instances of individual data points in the California Data Exchange records that were multiple orders of magnitude larger than the rest of the data and the dam storage capacity. All instances of these point errors are reported in the *agency_attributes.csv* of *ResOpsUS*.

## Data Records

**Fig. 2:**
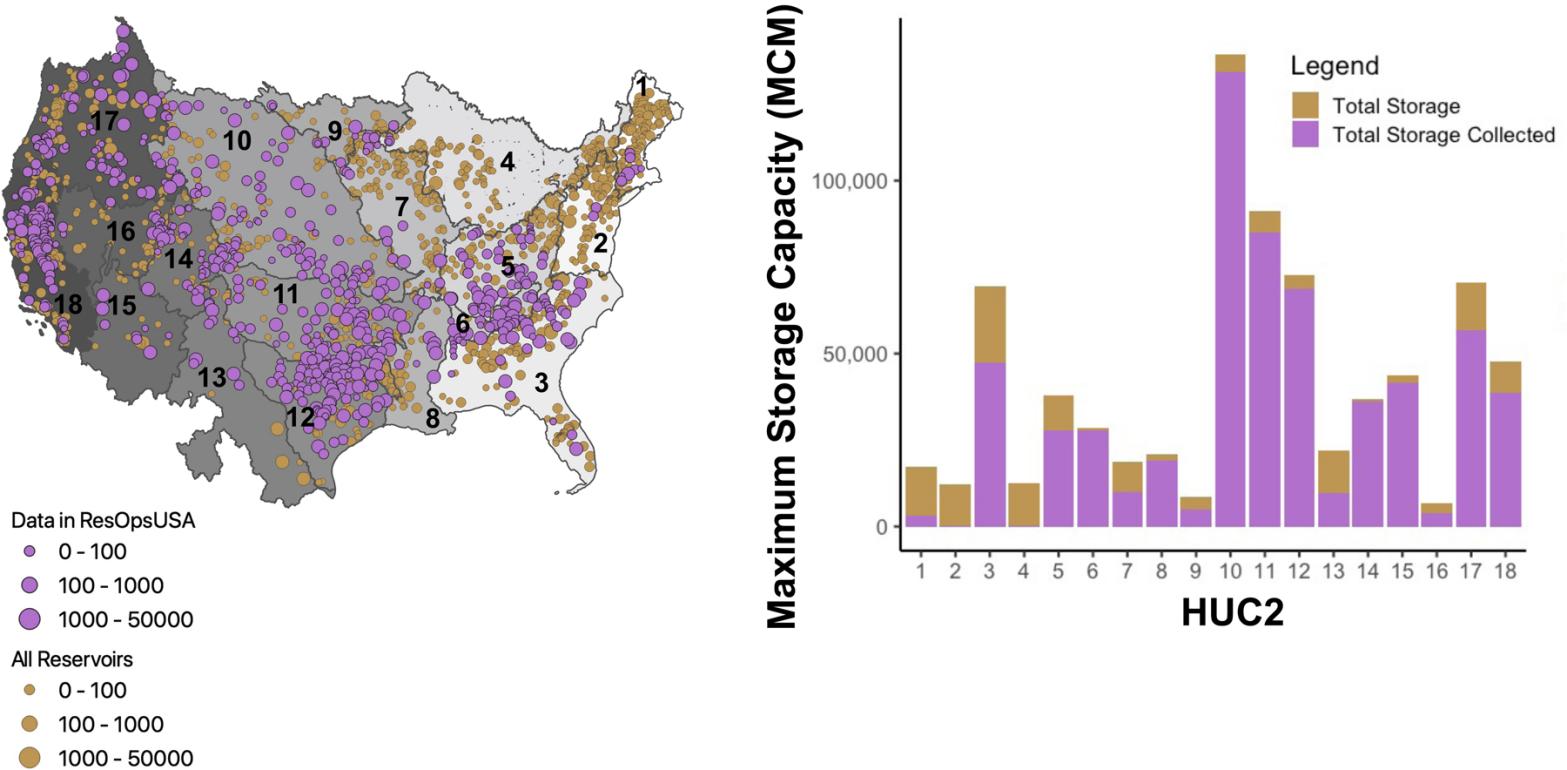
(**a**) Map of data coverage for the ResOpUS dataset (purple) compared to all of the large dams in the GRanD database (brown). All dams are scaled by size (maximum storage capacity in million cubic meters (MCM)) to demonstrate that ResOpUS contains the majority of dams greater than 1,000 MCM. (Note that the threshold for large dams in GRanD is dams higher than 15 meters or with storage >10 MCM, therefore there are some smaller storage dams in this map. However, this is not including all of the smaller storage structures which are not included in GRanD). (**b**) Data completeness per variable for all the dams in ResOpsUS the amount of total storage covered per HUC2 in million cubic meters (MCM). HUC1 HUC6 sit in the Eastern US HUC7 - HUC12 encompass the Midwestern US, and HUC13 – HUC18 encompass the Western US.

*ResOpsUS* is the first multi-agency, standardized, national dataset of historical reservoir observations across CONUS^23^. The dataset contains data for 679 dams in United States and 9 dams in Canada. Figure 2a maps the locations of the *ResOpsUS* reservoirs compared to all of the large reservoirs in the GRanD database. *ResOpsUS* covers observations for 87% of the 153 reservoirs in the US with storage capacities greater than 1000 MCM and 34% of the total storage of large dams with storage capacities greater than 10 MCM in the US. 653 of the reservoirs in *ResOpsUS* include daily storage records, 519 dams have daily outflow records, 321 have daily inflow records, 506 dams have daily elevation records, and 46 dams have daily evaporation estimates.

Figure 2b, shows the relative coverage by storage volume aggregated by HUC2 in million cubic meters (MCM). The HUC2s with the largest amount of storage (3, 5, 6, 8, 10, 11, 12, 14, 15, 16, 17, 18) all have over 50% of their storage covered in *ResOpsUS*. The HUC2s regions that are lacking storage coverage (2 and 4) correspond to the least amount of storage in CONUS, and are less likely to hold large dams (storage capacity greater than 1,000 MCM). Additionally, regions 2 and 4 have 288 (MCM) and 365 (MCM) total storage covered, so while they are lacking in data, they are not completely empty.

Spatial trends in coverage reflect trends in reservoir size across the US (Fig. 2a). The western U.S. generally has better coverage than the Northeast and Midwest. These regions generally have less coverage because many of the dams are smaller. Smaller dams are more likely to be included in the dataset when they are managed by federal agencies such as the Bureau of Reclamation (BOR) or the Army Corps of Engineers (ACE) (Fig. 1). Small dams that are independently operated (as is more common in these areas) are not covered in this dataset because we focused on gathering data for large dams. The large number of dam operators in the Great Lakes Region (HUC4) and the Eastern United States (HUC2) combined with the fact that most reservoirs are less than 25,000 MCM in these regions contributed to the lack of data acquired.

**Fig. 3:**
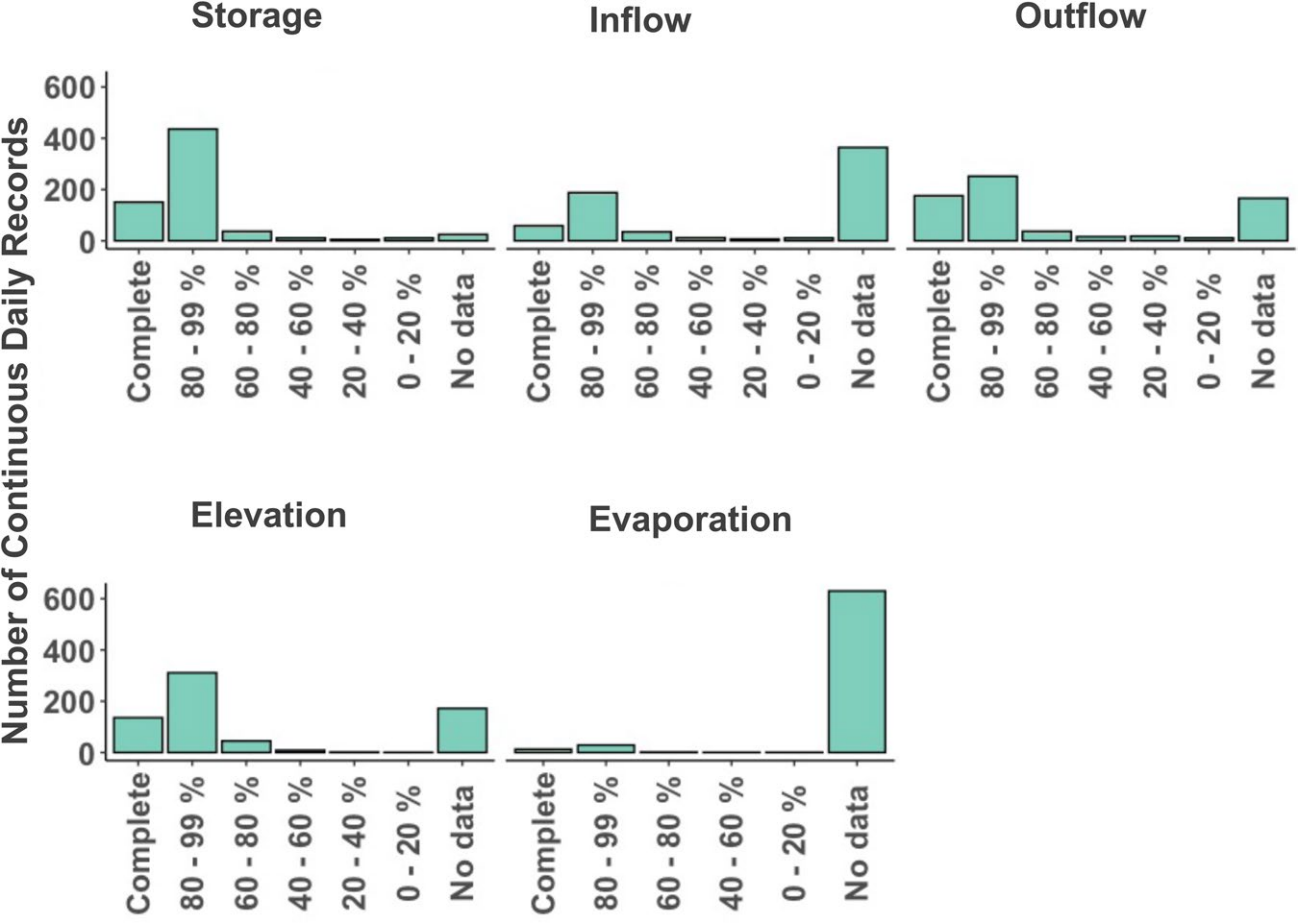
Data completeness histogram for ResOpsUS. All panels have number of records on the y axis and have been binned by percent completed for data starting at 1980 and ending in 2020. Panel a corresponds to storage. Panel b corresponds to inflow. Panel c corresponds to outflow. Panel d corresponds to elevation. Panel e corresponds to evaporation.

In all cases the total period of record for a reservoir was requested; however, in some cases the data were limited to the most recent years of operations. In some cases earlier operations were not available electronically, or there were gaps in the middle of the record due to data collection issues. We accepted available data even if the complete period of record was not covered and in these cases gaps are denoted by “NA” values in the dataset. Our observations have an average starting year of 1974 and an average ending year of 2020, which corresponds to approximately 40 years of data. There are also differences in the variables provided by each dam operator. Here too, we accepted all variables that were available and do not limit ourselves to only those dams featuring data for all variables. Figure 3 depicts the completeness of each requested variable in the database by variable. Storage and release have the best coverage, each with over 400 dams (Fig. 3, panels a and c). Inflow (Fig. 3, panel b) follows closely behind with 300 dams, but is lacking entirely in 47% of cases. When available, evaporation and elevation were added to the dataset, but these variables were not available as often (Fig. 3, panels d and e). In most cases either elevation or storage were provide. Storage was provided for almost all the dams. Evaporation data were generally only provided for reservoirs in the Bureau of Reclamation dataset(Fig. 3, panel e).

**Fig. 4:**
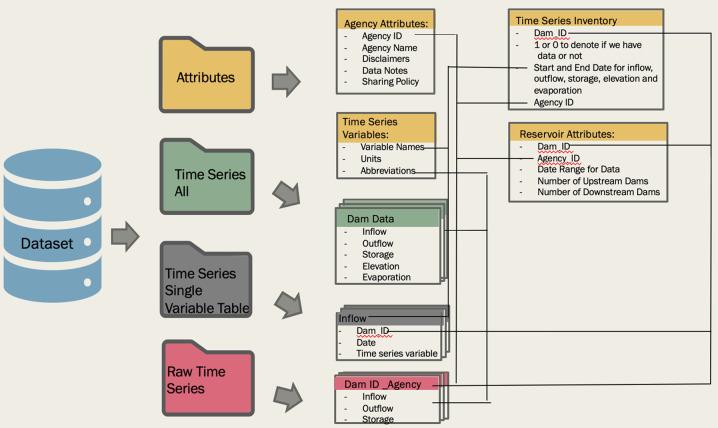
Schematic of the organized ResOpsUS dataset.

This organized database, as depicted in Fig. 4, contains four main folders: *attributes, time_series_all, raw_time_series,* and time_series_single_variable-table. The *time_series_all* folder contains individual CSV files for each dam that contain all the direct observations. All dams are organized using a unique ID (Dam_ID) that matches the GRanD dam id (Lehner *et al*., 2011). All time series files are organized by Dam_ID. In cases where we had data for one dam from multiple agencies, we chose the data with the most complete and longest period of record in the main time series folder. The duplicate files are located in raw_time_series folder labeled with the Dam_ID and Agency_ID. For instances where the data was not sourced online, JCS is listed to note where files were gathered via survey. We have also included single variable tables for storage, inflow and outflow with columns for all the Dam_IDs. The *attributes* folder provides information on the agencies that provided data for each dam (*agency_attributes*), the reservoir characteristics (i.e. dam height, location, storage capacity all in the *reservoir_attributes*) as well as an inventory of all the available time series data and date ranges (*timeseries_inventory*). Consistent variable names and units were used throughout the dataset and are described in the *time_series_variables*.

All data files are hosted on *Zenodo*. This repository is open access, and the review process for the stable DOI ensures that the data is in a reusable and consistent format. The stable DOI for ResOpsUSv1.1 is 10.5281/zenodo.5893641^23^.

## Technical Validation

*ResOpsUS* is the most comprehensive dataset of United States historical reservoir operations gathered to date. Therefore, there are no comparable datasets available for direct validation. To evaluate the data in *ResOpsUS*, we compared statistics of storage and outflow records to the existing static reservoir attributes dataset GRanD^2^. We use these comparisons to determine whether the observations are consistent with what we would expect based on reservoir properties.

We compare to two properties reported in the GranD database

1. Maximum storage capacity: This is the maximum volume of water that may be held in storage. The storage capacity values in GRanD came from a variety of sources such as the National Anthropogenic Barriers Dataset (NABD), the National Inventory of Dams (NID) and agencies^1,24^. In cases where, minimum, normal and maximum storage values were not clearly differentiated, they chose the largest reported value to be the maximum storage capacity, the middle value to be the normal storage capacity and the smallest value to be the minimum storage capacity. If only one value was reported this value was used for both maximum and normal storage capacity.
2. Average annual discharge: This discharge value at the location of the dam. These originally come from the flows simulated by the WaterGap model using the HydroSHEDS flow routing scheme and uses the WaterGAP^25^. It should be noted here that these flows are naturalized and are not intended to reflect reservoir releases.

**Fig. 5:**
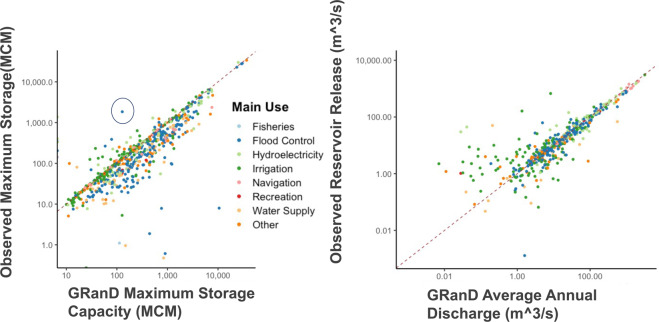
(**a**) Comparison between storage capacity in GRanD and the maximum observed storage values in ResOpsUS. (**b**) Comparison of average observed reservoir releases from ResOpsUS vs the average annual discharge from GRanD. The dashed line denotes the one-to-one line of perfect agreement between the two datasets.

Figure 5a compares the GRanD maximum storage capacity to the observed maximum storage volume. We expect that our storage values will be lower than GRanD storage capacities as we are evaluating actual transient storage (i.e. the water actually in the reservoir) rather than a static reservoir capacity (i.e. the total volume it can hold). Still this comparison can reveal potential data inconsistencies, such as instances where observed storage greatly exceeds reported capacity. Overall we see good agreement between *ResOpsUS and GRaND.* The majority of the reservoirs in *ResOpsUS* feature periods where the the maximum operational storage value is at or just below maximum operating capacity. It should be noted that no values were modified or removed in the ResOpsUS. However, large outliers are flagged in a column in the *agency_attributes.csv* file. Please note that we have not created an extensive list and have only denoted the inconsistencies that are orders of magnitude off from the GRanD values.

The coloring by primary reservoir usage indicates that there is a close match between observed maximum storage and reported maximum storage capacity for irrigation reservoirs, while for flood control reservoirs, which are operated with a significant buffer to capture flood water, the reported capacity is generally larger than the observed maximum storage, as expected. Cases where maximum storage levels exceed capacity may represent reservoirs that are spilling, or it could indicate an error in either the reported storage value in ResOpsUS or the GRanD storage capacity. In many cases, the differences between *ResOpsUS* and GRanD are small, but there are some large inconsistencies that should be noted. For example, the three main outliers (circled in blue) depict dams where the maximum observed storage in *ResOpsUS* greatly exceeds the maximum storage capacity in GRanD. In these cases, the inconsistencies are caused by a small number of point daily observations that skew the largest storage values. These appear to be small errors in the observation dataset caused by flooding conditions along the river systems. These datapoints are flagged in the Agency_Attributes file in the database.

Next, we compare the average annual discharge from GRanD to the observed reservoir releases from the period of 1971–2000 (Fig. 5b). Note, that as described above the GRanD flow values are simulated natural flows from the WaterGAP model, not stream gauge observations, and they do not include management. As such, we expect to see significant differences between ResOpsUS and GRanD especially where diversions and upstream consumption significantly alter flows. Additionally, it should be noted that the WaterGAP model is at a relatively coarse global resolution which can lead to discrepancies with the point observations provided in ResOpsUS especially for reservoirs with smaller drainage areas. Still, we provide these comparisons as a general illustration of agreement with simulated flows. As shown in Fig. 5, observed reservoir releases agree more closely with natural discharge for larger flows. It should be noted that the log scale on this plot does accentuate differences in the smaller flow. However, we would also expect to see larger discrepancies for smaller reservoirs which may have drainage areas not as well represented in the global model. Additionally, the natural flow estimations are closer to observed releases for flood control reservoirs which are mainly located in the South-eastern United States (Fig. 1).

**Fig. 6:**
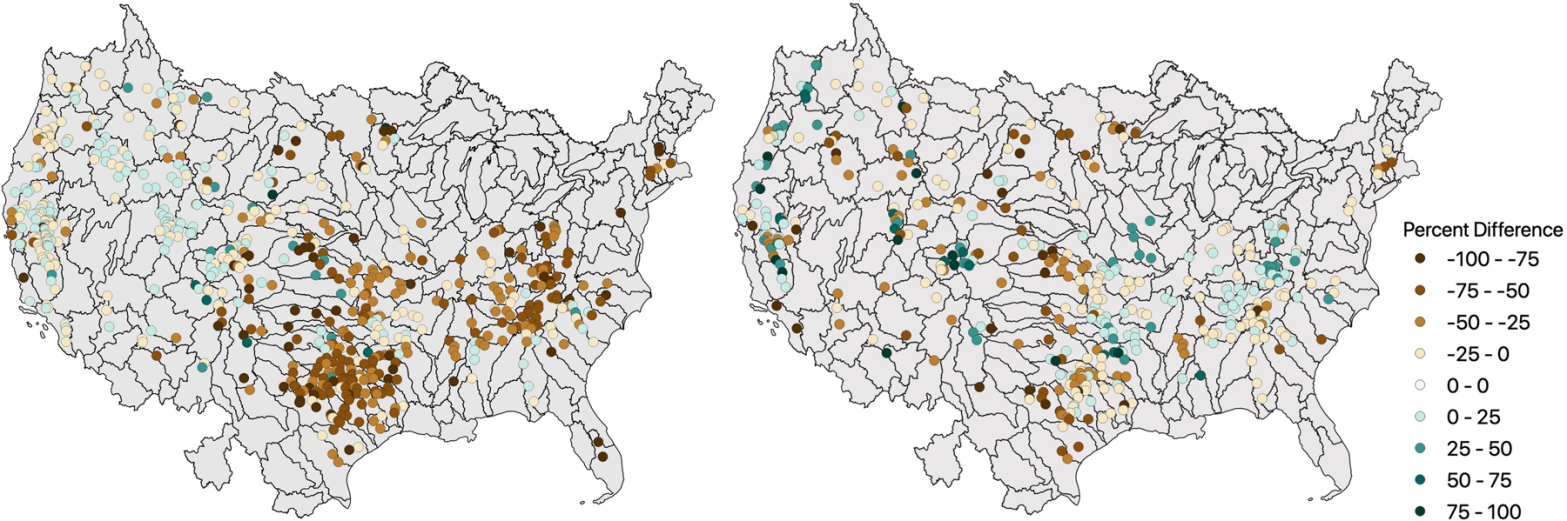
Spatial patterns in percent differences storage (**a**) and long-term average release (**b**) between ResOpsUS and GRanD. The top map (**a**) depicts the percent difference between the maximum storage capacity and the observed maximum storage. The bottom map (**b**) depicts the percent difference between the natural streamflow derived from HydroSHEDS and WaterGAP in GRanD and the observed average reservoir releases in ResOpsUS.

Figure 6 maps spatial differences in storage and flow between ResOpsUS and GRanD. The largest percent differences occur in the Southeastern and Midwestern US. This directly corresponds to areas that have a large number of flood control reservoirs whose storage capacity will vastly exceed the amount of water normally in storage in these regions. Smaller differences occur in the Western United States where water supply and irrigation reservoirs are more prevalent. This largest percent differences in discharge are seen mainly in the western United States where water consumption for irrigation is relatively high. In the southeast, where the dams are typically used for navigation and hydropower, we see smaller percent differences.

## Usage Notes

Before using the data, please turn your attention to the methods section of this paper as well as Fig. 4 to observe how the dataset is organized. This organization will be useful in determining how best to filter and compile the select data you would like.

This dataset can easily be merged with GRanD using the Dam_ID. Additionally, the STATE and AGENCY_CODE columns can be used to filter out data for individual states or data from different agencies in order to compile the data for the domain you are looking at. Additionally, users interested in reservoir operations should refer to Turner *et. al.*, 2021 for reservoir operating policies derived from ResOpsUS^22^.

## Data Availability

Data processing was done in R using the tidyverse packages. Data came from a variety of sources in many different formats most processing had to be done on a reservoir by reservoir basis. The dataset is currently hosted with a static DOI (see below) on *Zenodo* and is 299.3 MB. DOI link for dataset: 10.5281/zenodo.5893641^23^.
